# Community Structure of Arbuscular Mycorrhizal Fungi in Rhizospheric Soil of a Transgenic High-Methionine Soybean and a Near Isogenic Variety

**DOI:** 10.1371/journal.pone.0145001

**Published:** 2015-12-14

**Authors:** Jingang Liang, Fang Meng, Shi Sun, Cunxiang Wu, Haiying Wu, Mingrong Zhang, Haifeng Zhang, Xiaobo Zheng, Xinyuan Song, Zhengguang Zhang

**Affiliations:** 1 Department of Plant Pathology, College of Plant Protection, Nanjing Agricultural University, and Key Laboratory of Integrated Management of Crop Diseases and Pests, Ministry of Education, Nanjing, China; 2 Development Center of Science and Technology, Ministry of Agriculture, Beijing, China; 3 The National Key Facility for Crop Gene Resources and Genetic Improvement (NFCRI), MOA Key Laboratory of Soybean Biology (Beijing), Institute of Crop Science, The Chinese Academy of Agricultural Sciences, Beijing, China; 4 Nanchong Academy of Agricultural Science, Nanchong, China; 5 Agro-biotechnology Research Institute, Jilin Academy of Agriculture Sciences, Changchun, China; Estación Experimental del Zaidín (CSIC), SPAIN

## Abstract

The use of transgenic plants in agriculture provides many economic benefits, but it also raises concerns over the potential impact of transgenic plants on the environment. We here examined the impact of transgenic high-methionine soybean ZD91 on the arbuscular mycorrhizal (AM) fungal community structure in rhizosphere soil. Our investigations based on clone libraries were conducted in field trials at four growth stages of the crops each year from 2012 to 2013. A total of 155 operational taxonomic units (OTUs) of AM fungi were identified based on the sequences of small subunit ribosomal RNA (SSU rRNA) genes. There were no significant differences found in AM fungal diversity in rhizosphere soil during the same growth stage between transgenic soybean ZD91 and its non-transgenic parental soybean ZD. In addition, plant growth stage and year had the strongest effect on the AM fungal community structure while the genetically modified (GM) trait studied was the least explanatory factor. In conclusion, we found no indication that transgenic soybean ZD91 cultivation poses a risk for AM fungal communities in agricultural soils.

## Introduction

Modern agricultural biotechnology and genetic engineering have allowed the development of crops with improved properties, and global biotech crop hectarage has increased from 1.7 million hectares in 1996 to 181.5 million hectares in 2014 [1]. The field application of genetically modified plants (GMPs), however, might have undesirable consequences on the surrounding ecosystem, such as plant-beneficial soil microorganisms. Non-target organisms could be affected (a) by the products of the transgene itself; (b) by metabolites of the transgene products; or (c) by interaction with an altered plant phenotype [2,3]. Arbuscular mycorrhizal fungi (AMF) represent a potential key non-target organism to be monitored in studies on the ecological effects of GM crops [4]. Several previous studies on the impact of GMPs monitoring changes in the diversity of whole rhizosphere-associated fungal and bacterial communities have revealed either minor or no effect [3,5,6]. However, the total number of studies about the impacts of transgenic crops on AMF is rather low [7], especially about certain traits such as enhanced nutrition (e.g., increased methionine content).

Plant-beneficial microorganisms are widely recognized as a crucial natural component of the fertility for agricultural soils. AMF are known to be involved in promotion of plant growth and health, and they are more sensitive to changes in the host plant than free-living soil fungi [7]. AMF, which belong to phylum Glomeromycota, can form mutualistic symbioses with the majority of land plants, including many crops (e.g., soybean) [8]. Verbruggen et al. [9] found that AMF comprise a large portion of soil fungal communities, and it should not be ignored in environmental risk assessment of transgenic crops. Previous studies have shown that GM plants can alter AM fungal development (e.g., delay the colonization, reduce pre-symbiotic hyphal growth, and affect the regular development of appressoria), suggesting that there is a potential for adverse transgenic plant-induced impacts on AMF [10,11]. Furthermore, Liu suggested more work is needed to be done to elucidate the impacts of GM crops on AM fungi [12].

As the methionine (Met) content limits the nutritional value of soybean, GM soybean ZD91 was engineered to enhance its Met content and increase its nutritional value [13]. Transgenic soybean ZD91 was reported to have no significant impact on rhizosphere bacterial community structure [5], but so far, the effect of transgenic soybean ZD91 on plant-beneficial AMF (which is a relevant indicator of microbe-plant symbioses in nutrient acquisition) is unknown [3]. Transient effects of GM crops (e.g., *Bt* maize, starch modified potatoes, and herbicide tolerant soybean) on soil AM fungal community structure or root colonization have been reported [10,14–16]. As plants vary naturally in their AMF-hosting ability, the GM trait in plants might, in some cases, alter their relationship with AMF [7]. So whether transgenic soybean plants with altered content of methionine will affect AMF?

Filion [17] suggested that risk assessment studies on the effect of GMPs should be conducted under field conditions over at least two years. Hannula et al. [16] suggested that it is important to consider the phenological growth stages of plants when assessing the effect of GM crops on the environment. Previous studies usually focused on one year or one growth stage, so the question remains: is there any effect of GM variety on diversity of the soil microbes over multiple years [18]? In our study, potential effects of transgenes introduced into soybean on AMF assessed by comparing communities found in the rhizosphere of transgenic and non-transgenic soybeans at various stages of crop growth and various planting years. Four growth stages of transgenic high-methionine soybean line ZD91 and its parental isoline ZD each year from 2012 to 2013 were included in this study allowing us to determine the long-term (years) and short-term (within growth season) effects of the transgenic high-methionine soybean on AMF community structure dynamics.

## Materials and Methods

### Ethics statement

This study was approved by the Ministry of Agriculture of the People’s Republic of China and the genetically modified organisms safety team of Nanjing Agricultural University, China. The land was not privately owned or protected in any ways, and the field studies did not involve any endangered or protected species.

### Soybean Cultivars

Transgenic soybean cultivar (ZD91) contains the *Arabidopsis* cystathionine γ-synthase (*AtD*-*CGS*) gene which has been introduced artificially into the soybean cultivar Zigongdongdou (ZD) using *Agrobacterium*-mediated transformation, and exhibits a high content of methionine in the seeds. This gene was expressed under the control of a seed-specific promoter legumin B4. Briefly, ZD is a soybean cultivar developed via the conventional breeding process, and it is an unmodified original plant. There are no significant differences on other agronomic traits (e.g., plant height, pod number per plant, seed number per plant, 100-seed weight, and yield) between transgenic plant (ZD91) and wild type line (ZD) [13].

### Field setup and sampling

This study was performed at Nanchong (30°48′N, 106°04′E), Sichuan Province, China, in which a completely randomized block design was set out in two consecutive growing seasons (2012–2013, i.e., the third and fourth years of the experiment) of soybean in the same field. The mean monthly temperature and rainfall during the experiment were provided by Nanchong Meteorological Bureau (Table 1). Each cultivar had four randomly distributed blocks, and the field was under standard agricultural practice. Irrigation, fertilization and field management were implemented according to the conventional methods in the area. Rhizosphere soil samples were collected in both years at four different growth stages [seedling stage (SS), flowering stage (FS), pod-setting stage (PS), and maturity-setting stage (MS)]. Collection of soil samples using sterile techniques, the samples were collected as described previously [5]. In brief, five soybean roots per block were removed from the soil by hand and taken to the laboratory. Loosely adhering soil on the roots was shaken off and discarded, and the more tightly adhering soil was then brushed off and collected. Rhizosphere soils from the five plants per block were mixed and used as a composite sample [5]. The rhizosphere soil samples were sieved using a 20-mesh sieve (aperture size is 830 μm) and then stored at -20°C until further use [19]. The main physical and chemical properties of the soil are provided in Table 2. Soil pH was measured with a pH meter (PB-10, Sartorius) using a 1:2.5 soil-to-water solution [19]. The concentrations of total carbon and nitrogen in rhizosphere soil were determined by Vario MICRO cube (Elementar, Germany) [5].

**Table 1:** The mean monthly temperature (°C) and rainfall (mm) during the experiment. SS: seedling stage; FS: flowering stage; PS: pod-setting stage; MS: maturity-setting stage.

		July/SS	August/FS	September/PS	October/MS
2012	Temperature	27.13	29.27	22.53	18.07
	Rainfall	83.10	67.20	69.13	15.90
2013	Temperature	29.37	29.37	22.70	19.33
	Rainfall	61.90	45.40	32.63	18.97

**Table 2:** pH, total C and N contents (%), library coverage estimation (%), root colonization (%) and AMF diversity in rhizosphere soil (means ± standard errors) (*P* < 0.01). SS: seedling stage; FS: flowering stage; PS: pod-setting stage; MS: maturity-setting stage.

		SS		FS		PS		MS	
		ZD	ZD91	ZD	ZD91	ZD	ZD91	ZD	ZD91
2012	pH	8.40 ± 0.02	8.43 ± 0.01	8.40 ± 0.01	8.33 ± 0.07	8.36 ± 0.02	8.30 ± 0.06	8.19 ± 0.02	8.22 ± 0.02
	Total C	1.46 ± 0.08	1.53 ± 0.04	1.42 ± 0.10	1.59 ± 0.09	1.69 ± 0.11	1.74 ± 0.15	1.63 ± 0.11	1.58 ± 0.12
	Total N	0.08 ± 0.01	0.07 ± 0.01	0.09 ± 0.02	0.08 ± 0.01	0.08 ± 0.02	0.09 ± 0.01	0.08 ± 0.01	0.07 ± 0.01
	Coverage	93.62 ± 5.06	95.71 ± 1.70	91.13 ± 1.54	88.75 ± 4.16	93.57 ± 1.85	92.27 ± 2.43	94.52 ± 1.52	93.97 ± 4.16
	Root colonization	6.00 ± 4.58	9.00 ± 3.61	56.00 ± 5.57	57.33 ± 4.62	51.00 ± 3.61	56.33 ± 0.58	61.67 ± 4.39	55.42 ± 4.73
	Shannon index	1.85 ± 0.32	1.78 ± 0.26	1.74 ± 0.37	1.75 ± 0.23	1.54 ± 0.22	1.88 ± 0.28	1.54 ± 0.19	1.64 ± 0.10
2013	pH	8.21 ± 0.04	8.20 ± 0.03	7.93 ± 0.04	7.89 ± 0.07	8.08 ± 0.05	8.08 ± 0.09	8.14 ± 0.05	8.17 ± 0.08
	Total C	1.81 ± 0.01	1.78 ± 0.07	1.75 ± 0.04	1.76 ± 0.03	1.74 ± 0.03	1.72 ± 0.04	1.71 ± 0.02	1.71 ± 0.02
	Total N	0.09 ± 0.01	0.09 ± 0.01	0.08 ± 0.01	0.08 ± 0.01	0.09 ± 0.01	0.09 ± 0.01	0.08 ± 0.01	0.08 ± 0.01
	Coverage	81.25 ± 7.50	81.77 ± 8.24	90.40 ± 8.31	94.05 ± 4.22	71.38 ± 15.86	76.88 ± 4.63	70.06 ± 12.12	67.73 ± 11.06
	Root colonization	12.33 ± 6.11	13.00 ± 8.54	60.33 ± 0.58	58.00 ± 3.46	54.33 ± 6.03 A	31.67 ± 5.51 B	65.42 ± 3.82	61.25 ± 2.17
	Shannon index	1.76 ± 0.60	1.88 ± 0.34	1.33 ± 0.61	0.77 ± 0.47	2.40 ± 0.63	2.21 ± 0.52	2.69 ± 0.21	2.67 ± 0.62

### Root Colonization

Root colonization is often used as an indicator of symbioses development [20]. 30 randomly chosen roots were carefully washed, cut into 1 cm long segments, cleaned in 10% KOH at 90°C for 20 min, acidified in 2% HCl for 5 min, and stained with 0.01% acid fuchsin [21]. The extent of mycorrhizal colonization was calculated according to the grid-line intersection method [22].

### Soil DNA extraction and PCR

The soil DNA was extracted from the rhizosphere soil samples by employing the MoBio PowerSoil DNA Isolation Kit (MoBio Laboratories, Inc., USA) with the method recommended by the manufacturer. DNA concentrations were quantified by using a NanoDrop 1000 Spectrophotometer (Thermo Scientific, USA) according to the manufacturer’s protocol. Partial SSU rRNA gene fragments were amplified using nested PCR with the universal eukaryotic primers NS1 and NS4 [23]. PCR was carried out in a final volume of 25 μL [each reaction for PCR consisted of 2.5 μL 10 × PCR buffer (Mg^2+^ Plus), 2 μL 2.5 mM dNTP, 0.25 μL each of 20 μM primers, and 0.125 μL rTaq DNA polymerase (5 U μL^-1^), to which were added 0.25 μL template DNA and then sterile distilled water to a final volume of 25 μL] and the cycling conditions were as follows: initial denaturation at 94°C for 5 min, then 30 cycles with denaturation at 94°C for 30 s, annealing at 56°C for 30 s, followed by elongation at 72°C for 90 s. The cycle was finalized by elongation at 72°C for 10 min. The PCR products were further amplified in the second step with the AM fungi general primer pair AML1 and AML2 (PCR system: see above; PCR conditions: 94°C for 5 min, then 30 cycles of 30 s denaturation at 94°C, 30 s primer annealing at 64°C and 1 min extension at 72°C, followed by a final extension period of 10 min at 72°C) [23]. Reaction yields were estimated by using a 1% agarose gel containing ethidium bromide. The PCR products obtained from DNA extracted from ZD and ZD91 soil samples were used to construct SSU rRNA gene libraries.

### Cloning and sequencing

The PCR products of the expected size (~800 bp) were purified using the TaKaRa MiniBEST Agarose Gel DNA Extraction Kit (TaKaRa, Japan) and were cloned into pMD19-T Vector (TaKaRa, Japan). The vector was transformed into *E*. *coli* strain DH5α, which were spread on agar plates and incubated overnight at 37°C. Inserts from 80 randomly selected clones in each resulting SSU rRNA gene library (4 blocks × 4 growth stages × 2 years × 2 cultivars = 64 libraries) were sequenced using M13-f47 and M13-r48 primers on ABI 3730 genetic analyzer. The obtained sequences were trimmed to remove the vector sequence, and sequence similarities were determined using the Basic Local Alignment Search Tool for nucleotides (BLASTn) sequence similarity search tool provided by GenBank [24]. Sequences were clustered at 97% sequence similarity with QIIME [25] and a representative sequence from each OTU was picked for downstream analysis. Taxonomic assignments of OTUs were performed by using QIIME in accordance with SILVA 115 [26]. Representative sequences of the clones generated in this study were deposited at the National Centre for Biotechnology Information (NCBI) GenBank database under the accession numbers KJ740816 to KJ740970.

### Statistical analysis

One-way analysis of variance and Duncan pair-wise comparisons (*P* < 0.01) were used to determine the minimum significant differences between soybean cultivars by employing SPSS version 17.0 for Windows [27]. In order to assess the efficiency of the clone library, the rarefaction curve was analyzed by the freeware program Analytic Rarefaction 1.3 of the Stratigraphy Lab, University of Georgia [28]. The percentage of coverage was calculated using Good’s coverage equation [29], this calculation estimates how well the clones accounted for the biodiversity. Principal component analysis (PCA) was performed in R to compare AMF community structure across all samples [30]. ADONIS differences were calculated by using the vegan package in R [31,32]. ADONIS implements a multivariate analysis of variances using distance matrices and function ADONIS can handle both continuous and factor predictors. We used Jaccard as a distance index and 10,000 permutations. ADONIS analysis between cultivar, growth stage and year and the interactions among them were provided. Variance partitioning analysis (VPA) was also used to determine the contributions of cultivar, growth stage and year, as well as interactions between them on the variation in a AMF community structure with Hellinger-transformed data [31,32].

AMF diversity was calculated using the Shannon (*H*) index [24] using clone numbers of each OTU in each sample as fungal abundances. Richness of a given sample is all OTU numbers in that sample.

## Results

### Main physical and chemical properties of the soil, mycorrhizal colonization and AMF diversity

Through the four different growth stages of each year, the soil pH fluctuated around 8.00, and there were no significant differences between soybean lines ZD and ZD91 (Table 2). The effects of the transgenic soybean on C and N concentrations in rhizosphere soil were examined, and there were no significant differences between ZD and ZD91 (Table 2). We found that there were no significant differences in the intensity of root colonization by AMF between ZD and ZD91 within the same growth stage in 2012 and 2013, except PS stage in 2013 (Table 2). In general, AMF colonization increased over time, and the highest colonization rate was evident at the maturity-setting stage. The AMF diversity, expressed by the Shannon (*H*) index, was similar for ZD and ZD91 within the same growth stage in 2012 and 2013 (Table 2).

### PCR amplification of AMF SSU rRNA gene sequences from soil and diversity analyses

**Fig 1:**
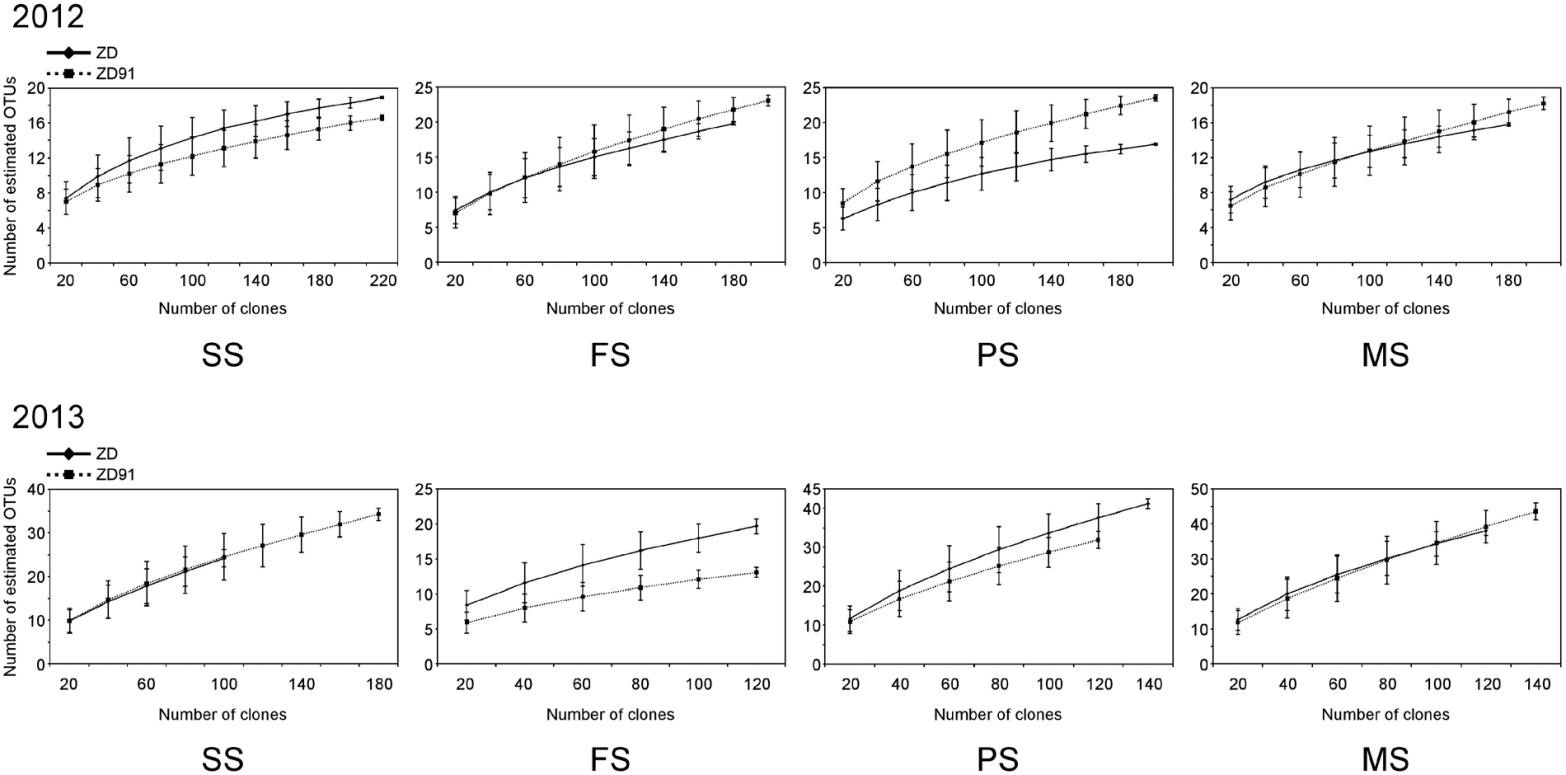
Rarefaction analysis. Rarefaction curve of the OTUs obtained from soybean rhizosphere soil of various cultivars. Nucleotide sequences with greater than 97% identity were referred as the same OTU. SS: seedling stage; FS: flowering stage; PS: pod-setting stage; MS: maturity-setting stage.

In addition to AMF root colonization levels, it is crucial to check whether the composition of AMF communities is altered by GM plants [9]. A total of 5,120 clones from 64 libraries were sequenced and screened for the presence of AMF DNA (80 clones per library). GenBank BLAST search indicated that 2,838 clones were from Glomeromycota. These 2,838 AMF sequences were grouped into 155 OTUs at a 97% similarity threshold (S1 Table). Based on the result of rarefaction curves (Fig 1) and library coverage value calculations (Table 2), the number of clones analyzed was able to identify the major AM fungi in rhizosphere soil.

**Fig 2:**
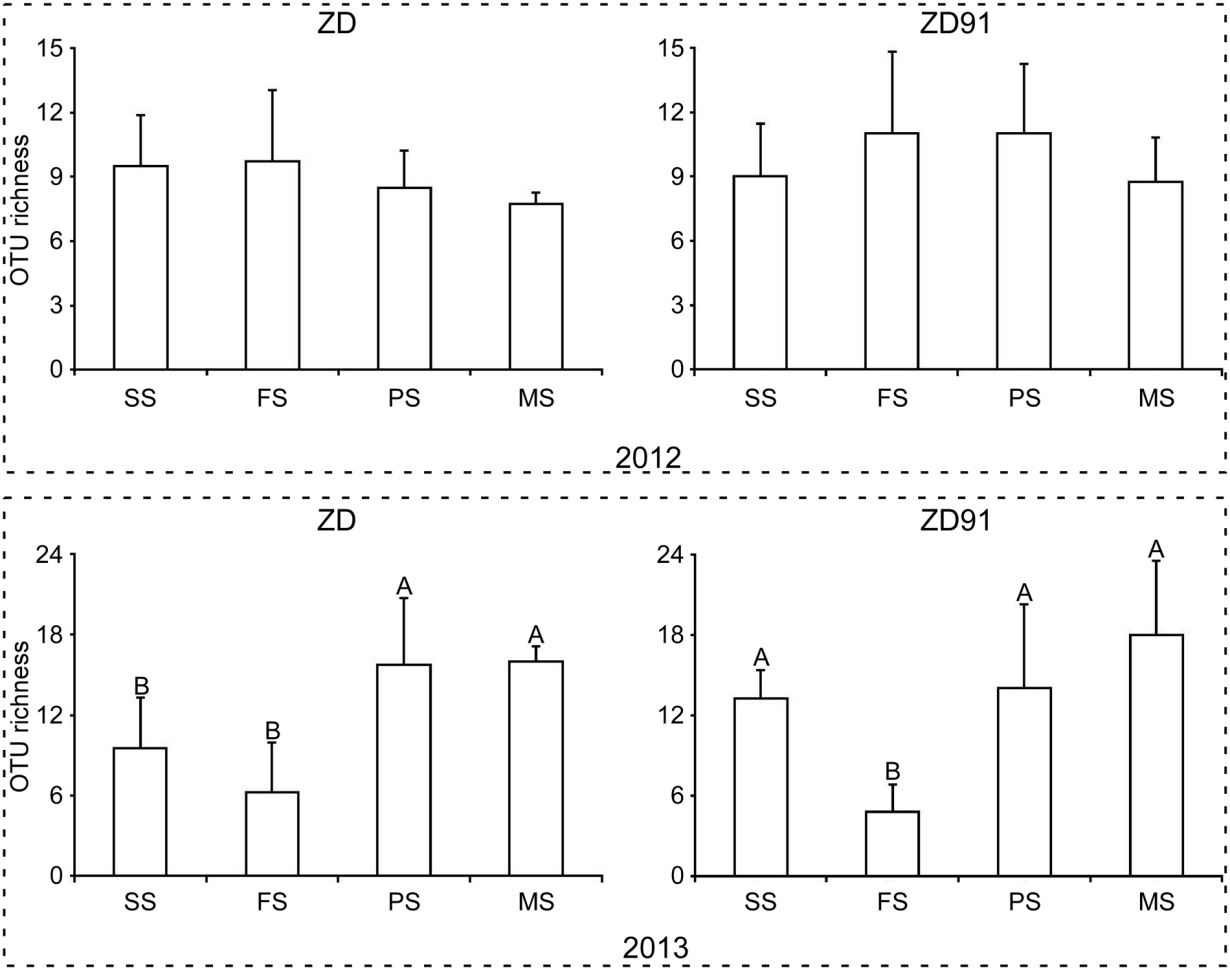
AM fungal OTU richness in soil. The letter above the column indicates significant differences in the richness of AM fungal OTUs among the four growth stages (*P* < 0.01). SS: seedling stage; FS: flowering stage; PS: pod-setting stage; MS: maturity-setting stage.

Of the 155 AMF OTUs present in rhizosphere soil, 96 (47.04% of total clone sequences) were found in ZD, 103 (52.96% of total clone sequences) in ZD91, and 44 OTUs were shared between the two. Of the 155 OTUs, 58 were detected in 2012, 138 were detected in 2013, and 41 OTUs were observed in both years. There were no significant differences between AM fungal OTU richness in ZD and ZD91 within the same growth stage in 2012 and 2013 (Fig 2). However, the growth stage had a significant effect on AM fungal OTU richness in 2013, but not in 2012. In 2013, the OTU richness in the seedling and flowering stages of ZD was significantly lower than that in the other two stages; in contrast, the OTU richness in the flowering stage was significantly lower than that in the other three stages in ZD91.

### AMF community structure

**Fig 3:**
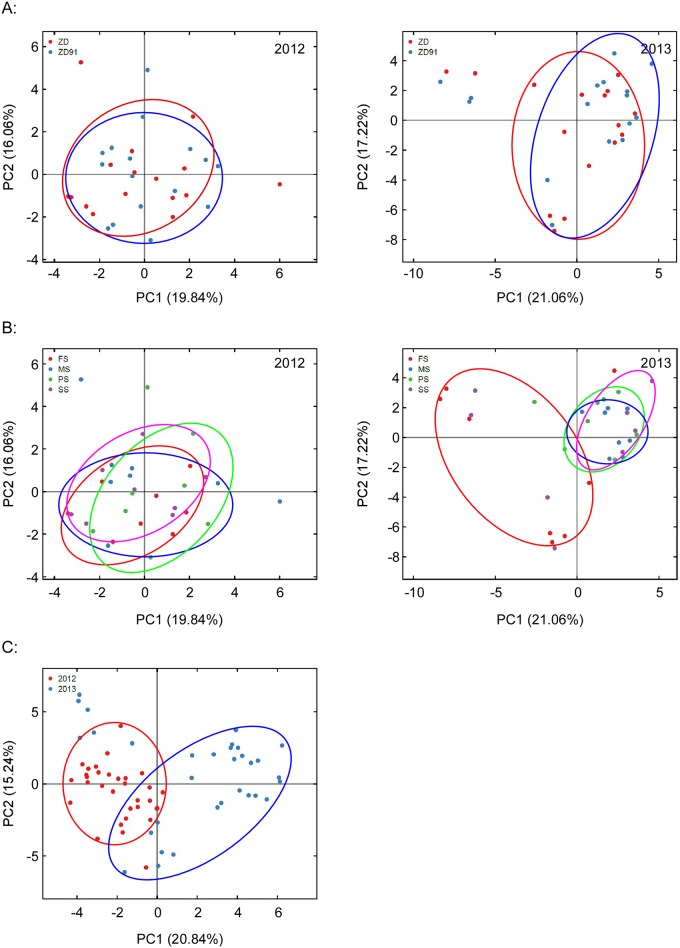
PCA of AMF community structure based on OTUs at a distance of 3% for individual samples. A: AMF community structure between cultivars. B: AMF community structure between growth stages. C: AMF community structure between years. The eigenvalues displayed on the diagram axes refer to the percentage variation of the respective axis. SS: seedling stage; FS: flowering stage; PS: pod-setting stage; MS: maturity-setting stage.

We applied PCA to compare AMF community structure between cultivar, growth stage and year (Fig 3). When the cultivar was examined as an explanatory variable, there were no significant differences found in the AMF community structure of ZD and ZD91 in the same year (Fig 3A), but the community structure were clearly distinct for different years (Fig 3C). In 2013, an effect of the growth stage was observed as the flowering stage was separated from the other stages, however, this effect was not seen in 2012 (Fig 3B). We also calculated ADONIS differences between cultivar, growth stage and year and the interactions among them (Table 3). According to ADONIS, the AMF community structure was most strongly influenced by year-to-year variation and growth stage.

**Table 3:** ADONIS analysis of effects of cultivar, growth stage, year, and the interactions between them on the AMF community structure in rhizosphere soil.

	F. Model	R^2^	Pr (> F)
Growth Stage	1.71	0.07	0.01
Cultivar	0.86	0.01	0.59
Year	9.10	0.12	0.00
Growth Stage × Cultivar	0.94	0.04	0.58
Growth Stage × Year	1.82	0.07	0.00
Cultivar × Year	0.74	0.01	0.76
Growth Stage × Cultivar × Year	0.87	0.04	0.70

### Linking the AMF community structure to cultivar, growth stage, and year

**Fig 4:**
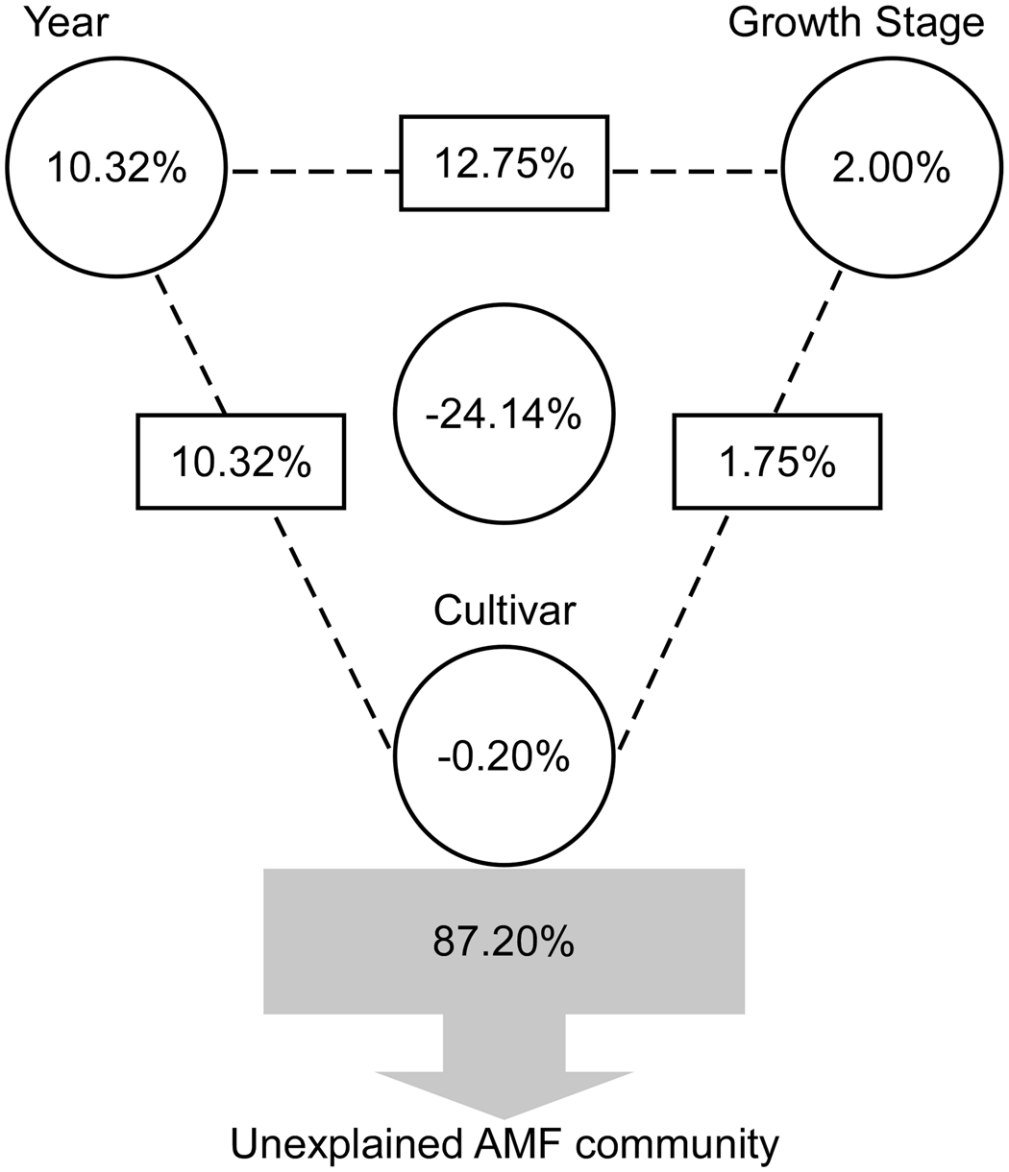
VPA of the AMF community structure. Effects of cultivar, growth stage, planting year, and the interactions between them on the AMF community structure. Circles on the edges of the triangle show the percentage of variation explained by each factor alone. The percentage of variation explained by interactions between two or three of the factors is shown as squares on the sides and as circle in the center of the triangle. The unexplained variation is depicted in square on the bottom.

To quantify the relative contributions of cultivar, growth stage, and year to the total AMF community structure based on OTU composition, VPA was performed and the variation in AMF community structure was partitioned among cultivar, growth stage and year, as well as the interactions between them. These variables explained 12.80% of the observed variation, leaving 87.20% of the variation unexplained (Fig 4). Growth stage explained small portions of the observed variation, which accounted for 2.00% (*P* = 0.22), while the year accounted for 10.32% (*P* = 0.04) of the total variation. The variation was mostly explained by interactions between year and growth stage, which accounted for 12.75%. The interactions between year and cultivar, and growth stage and cultivar, accounted for 10.32% and 1.75% of the variation, respectively. The VPA analysis combined with ADONIS analysis and PCA analyses revealed that the year was the most important factor in explaining the shifts in the AMF community structure, while the cultivar was not. Furthermore, the AMF community structure was marginally related to the growth stage.

## Discussion

The present study aims to assess the effect of transgenic soybean ZD91 on AM fungal community structure during two consecutive years (2012–2013). The results demonstrated that both plant growth stage and planting year impacted AMF community structure while the GM trait was the weakest explanatory factor. Our findings were consistent with those of others that evaluated the potential impacts of GM plants on AMF, indicating a transient or no impact of transgenic plants on the AMF [3,9,10,14–16,18,33–41], while three other studies have reported a significant effect of *Bt* maize on AMF [42–44]. Factors such as GM-parent, growth stage, field site, season, and cultivar were used to study the effects of GM crops on AMF [7]. Many studies revealed that the field site, the field season, the plant growth stage and year are important factors affecting AMF while GM-trait have transient or no effect [3,9,14,18,35,36,40,43]. Cotton et al. [45] found that the AM fungal community changes dramatically between years, and they were also suggesting that studies carried out during a single year may be difficult to interpret. In our study, the planting year was the largest explanatory factor, followed by plant growth stage (Table 3, Figs 2–4).

Factors such as annual variation and plant growth stage are more influential on AMF community structure than the GM trait [7]. Growth stage-related differences of AMF communities in rhizosphere soil can be explained by the interaction between the abiotic environment and the host phenology [18,46]. Although we have not measured root exudates in this research, there is already evidence that plant growth stage can affect root exudate fluxes which in turn affect soil microorganisms [47,48]. We also detected interesting differences between the planting years. There were no significant differences in average temperature (F = 0.07, *P* = 0.80) and rainfall (F = 1.21, *P* = 0.31) between those two years. However, there were significant differences in total carbon content of soil (F = 41.40, *P* < 0.01) and soil pH (F = 59.04, *P* < 0.01) between those two years. AMF are strongly affected by changes in soil characteristics [18]. Meyer et al. [3] found that different environmental conditions (e.g., soil properties) occurring in different field seasons considerably affected soil microbial community structure. These factors mentioned above might explain the differences in AMF community structure we observed between 2012 and 2013. Of the 155 AMF OTUs, 58 were detected in 2012, 138 were detected in 2013. It may explain why only 41 were shared between 2012 and 2013. Wu et al. [19] found that the diversity of fungi might be affected by various environmental factors, such as temperature, humidity, and light. And it was showed that the AM fungal community changes dramatically between years [45].

A generalized AMF life-history begins with colonization of a root [49]. Previous study has demonstrated the importance of AM fungal colonization to plant growth and survival [50]. And recent survey of the relationship between soybean and AMF has focused on evaluating AMF root colonization [51]. One common method to quantify AMF abundance is root colonization measurements [52]. Arbuscular mycorrhizal fungi colonize plant roots and deliver many essential nutrients to the plant [53]. Previous studies have suggested that the plant age is an important factor influencing the AMF colonization percentage [3,54,55]. Our present results also indicated that the plant growth stage clearly had the strong effect on AMF colonization, while GM status did not significantly affect AM root colonization, except PS stage in 2013 (Table 2). Wu et al. [19] showed that the inconsistencies in the soil at seeding time may lead to the differences between transgenic and non-transgenic plant. Similarly, Gosling et al. [55] found that sampling time had a significant influence on colonization in soybean. Meyer et al. [3] found that the proportion of roots colonized by AM fungi increased from young to mature plants. The lack of significant yearly changes in root colonization in our study (F = 0.01, *P* = 0.94) is similar to that of White and Weil [56] and Naghashzadeh et al. [57]. This is similar to the finding by Meyer et al. [3] that AM root colonization was not significantly affected by GM wheat introduced with the *Pm3b* mildew resistance transgene. Likewise, Powell et al. [14] did not observe significant effect of the transgenic glyphosate resistant soybeans on AMF root colonization. However, Cheeke et al. [43] found a reduction in AMF colonization in multiple *Bt* maize lines. There is general agreement that GM crops should be assessed on a case-by-case basis [2,58,59]. In this study, the Shannon (*H*) index showed no significant differences in AMF species diversity between ZD and ZD91 within the same growth stage in 2012 and 2013 (Table 2).

In general, the most frequently detected AMF species in soybean fields belong to the Glomeraceae, Gigasporaceae, Acaulosporaceae, Diversisporaceae, and Paraglomeraceae families [28,55,60,61]. Glomeraceae, Claroideoglomeraceae, Diversisporaceae, Paraglomeraceae, Acaulosporaceae, and Archaeosporaceae were found in the present study. The interaction between host phenology, soil characteristics and habitat can lead to the changes of AM [46]. We postulate that soybean variety, field site, and climate are the underlying factor differentiating AMF phylotypes in these studies.

In conclusion, our study examined the effects of a transgenic high-methionine soybean ZD91 on soil AMF community structure. The AMF community structure was strongly affected by natural variations in the environment related to planting years and soybean growth stages. Our results demonstrated that no significant differences in AMF community structure exist between high-methionine soybean ZD91 and its isoline ZD.

## Supporting Information

S1 Table Different OTUs for each sample. The samples were named in the following form: year-growth stage-cultivar-number of replicate. SS: seedling stage; FS: flowering stage; PS: pod-setting stage; MS: maturity-setting stage.
(XLS)
